# Proton vs Hyperarc™ radiosurgery: A planning comparison

**DOI:** 10.1002/acm2.13075

**Published:** 2020-11-05

**Authors:** A. Boczkowski, P. Kelly, S. L. Meeks, K. Erhart, F. J. Bova, T. R. Willoughby

**Affiliations:** ^1^ Department of Neurosurgery University of Florida Gainesville FL USA; ^2^ UF Health Cancer Center–Orlando Health Orlando FL USA; ^3^ dotDecimal Sanford FL USA

**Keywords:** HyperArc, planning, Proton radiosurgery

## Abstract

For many patients, stereotactic radiosurgery (SRS) offers a minimally invasive, curative option when surgical techniques are not possible. To date, the literature supporting the efficacy and safety of SRS treatment techniques uses photon beams. However, with the number of proton therapy facilities exponentially growing and the favorable physical properties of proton beam radiation therapy, there is an opportunity to develop proton therapy techniques for SRS. The goal of this paper is to determine the ability of clinical proton treatment planning systems to model small field dosimetry accurately and to compare various planning metrics used to evaluate photon SRS to determine the optimum beam configurations and settings for proton SRS (PSRS) treatment plans. Once established, these plan settings were used to perform a planning comparison on a variety of different SRS cases and compare SRS metrics between the PSRS plans and HyperArc™ (VMAT) SRS plans.

## 1 INTRODUCTION

Over the past two decades, there has been a fourfold increase in proton therapy centers built in the United States. With mechanical and technological advances, such as single room proton therapy units, it is possible for more institutions to introduce proton therapy into their daily regimen. Even with the large growth of proton therapy facilities in the past two decades, few centers treat intracranial lesions using single fraction SRS techniques. This is in part due to the widespread availability and experience with Gamma Knife units and linac‐based SRS systems,^1^ and in part due to the absence of any commercially available proton stereotactic radiosurgery systems.^2^

New technologies in proton therapy such as intensity modulated proton therapy raise potential treatment options for PSRS. However, the initial spot size of most scanning beam proton units range from 2.5 to 14.5 mm, depending on the energy of the proton beam.^3^, ^4^, ^5^ Wang et al. found, using Monte Carlo simulations, that a spot size less than 4.3 mm is required to achieve better scanning beam proton plans when compared to photon techniques.^6^ This small spot size requirement is the limiting factor of proton scanning beams, making it difficult to accurately and precisely deliver doses to very small lesions (i.e., 10 mm diameter) without any additional collimation in place.

Currently, all PSRS treatments are performed on passive scatter proton therapy units. One limitation for passive scatter proton PSRS is the time required to replace devices in the machine for unique beam angles. Many of these technological limitations are advancing and, in many cases, even manufacturing time for patient devices (apertures and compensators) can be minimized in order to cut down on the time required to generate a plan. Additionally, planning studies may provide insight into the correct number of beams required that will cut down on the number of fields needed to provide an optimal PSRS plan and eliminate the need to fractionate cases due to time constraints.^7^, ^8^

The purpose of this study was to perform a planning comparison between PSRS plans and HyperArc™ generated SRS plans. Before performing the planning study, the accuracy of the planning system to generate proton plans using small fields was tested. Once the limitations of the treatment planning system (TPS) were defined, a single patient plan was used to optimize planning parameters. Using those plan parameters, plans were created on a set of ten previously treated SRS patients using both HyperArc™ planning and PSRS planning techniques.

This study is divided into three sections: (a) Evaluate the accuracy of small field dosimetry in a clinically commissioned TPS for double scatter proton therapy. (b) Determine appropriate beam settings for double scattered PSRS. (c) Perform a treatment planning comparison study between PSRS and photon volumetric modulated arc therapy (VMAT) SRS treatment plans.

## 2 MATERIALS AND METHODS

### 2.1 Verification of TPS dosimetry for small fields

Proton therapy beams are specified in terms of range (d’) and modulation width (m’).The ICRU Report 78^9^ defines different terms that are used to describe proton therapy beams. Used in this work are the range defined as the water equivalent depth in g/cm^2^ of the distal 90% (d’_90_) of the dose relative to the dose at the middle of the flattened spread out Bragg peak (SOBP). The modulation width (m’_95_) for the Mevion S250 is defined as the water equivalent distance (g/cm^2^) between the distal 90% and proximal 95% and is noted as m’_95_ points on the SOBP. The distal dose falloff (DDF) is defined as the distance between the 80% to 20% penumbra on the distal end of the depth dose curve. The lateral penumbra (LP) is defined as the distance between the 80% and 20% of the dose falloff perpendicular to the beam axis. The d’_90_, m’_95_, DDF, and LP were compared between treatment planning system, film measurements, and water tank measurements.

Brass collimators with diameters equal to 1, 2, 3 and 4 cm were created for physical measurements of small field SOBP, and penumbra (both lateral and distal) for several ranges. Measurements in water were acquired using a PTW 3D water scanning system with a PTW PinPoint ion chamber (0.016 cm^3^) to ensure the detector was well placed in the center of the small field. Detector placement was also checked by taking profiles at multiple depths. To verify depth correction with the PinPoint chamber, SOBP depths were inter‐compared between the PinPoint chamber and a Markus plane parallel plate chamber for depth comparison using a standard calibration field size of 10 cm for protons reference. Scanning in all instances are performed by stepping the ion chamber in increments of 1 mm in the area of the distal edge of the spread out Bragg peak. Film measurements were acquired using EBT3 radiochromic film. A custom‐made solid water film phantom was developed to sandwich the film between two pieces of solid water. This phantom allowed for reproducible placement of the film relative to the surface for in‐beam measurements of depth dose. Measurements of nominal proton ranges 6.5 g/cm^2^, 9.5 g/cm^2^, and 11.5 g/cm^2^ each using the same modulation of 3 g/cm^2^. The middle of the SOBP was placed at the machine isocenter of 200 cm for all measurements. All measurements were taken on a Mevion S250 double scatter proton delivery system (Mevion Medical Systems, Littleton, MA).

The water and film depth dose measurements were compared to profiles extracted from simulated treatment plans created using a 3‐dimensional digital water phantom in the Pinnacle™ TPS (Philips Medical Systems, Madison, WI) using the same field size, air gap, range, and modulation as the physical measurements. This planning system had been previously clinically commissioned and used for planning patients treated on a Mevion S250 double scatter proton machine.

### 2.2 Optimizing PSRS planning parameters

Prior to the planning comparison, a single test case was used to determine the optimal planning parameters for the PSRS treatment plans. This included determining optimal beam number, whether to use a compensator or not, normalization isodose line, and determining if a set of fixed gantry angles could be used to optimize planning time.

To first determine the prescription isodose volume that yields the steepest dose gradient, a treatment plan using a single beam in a simulated 3D water phantom was created using a range of 9.5 g/cm^2^ and modulation of 3 g/cm^2^. The distal and lateral penumbra between the 80% and 20% isodose lines for different normalization values from 100% to 50% of maximum dose were measured and plotted to determine which normalization value that would yield the sharpest LP and DDF.

The optimal plan experiment was conducted to determine general settings to be used for planning PSRS cases. A simple, nominally spherical tumor volume for a patient previously treated for SRS was used for planning purposes. Due to the accuracy of patient positioning under SRS conditions and assuming similar accuracy would be implemented for PSRS, setup margins were assumed to be the same as would be implemented for linac‐based SRS and a zero setup margin from gross target volume (GTV) to clinical target volume (CTV) was used.^10^ For planning purposes, this corresponds to the lateral aperture margin and therefore no additional lateral margin was used for setup error, but for coverage purposes lateral margins of 0.5, 1, 2, and 3 mm margins were auto‐generated around the CTV to determine the optimal margin due to the penumbra of the double‐scatter proton beam.

The proximal and distal margin is used to account for range uncertainties and is defined as a percentage of the range plus a constant factor. Based on the literature the proximal and distal margins vary from institution to institution. Massachusetts General Hospital uses 3.5%*Range + 1 mm, the MD Anderson Proton Therapy Center uses 3.5%*Range + 3 mm, the Loma Linda University Medical Center uses 3.5%*Range + 3 mm, the University of Pennsylvania uses 3.5%*Range + 3 mm, and the University of Florida Proton Therapy Institute uses 2.5%*Range + 1.5 mm.^11^ For this study, range uncertainty of 2%*Range + 2 mm was chosen. For SRS targets, this results in adding approximately 4–5 mm to the prescribed Range compared to the depth of the target. The same margin is used on the proximal side of the SOBP which increases the overall modulation needed. Additionally, the Mevion S250 machine has a minimum modulation width of 2 cm, so for smaller targets there is additional range and modulation margin due to this constraint.

**Fig. 1:**
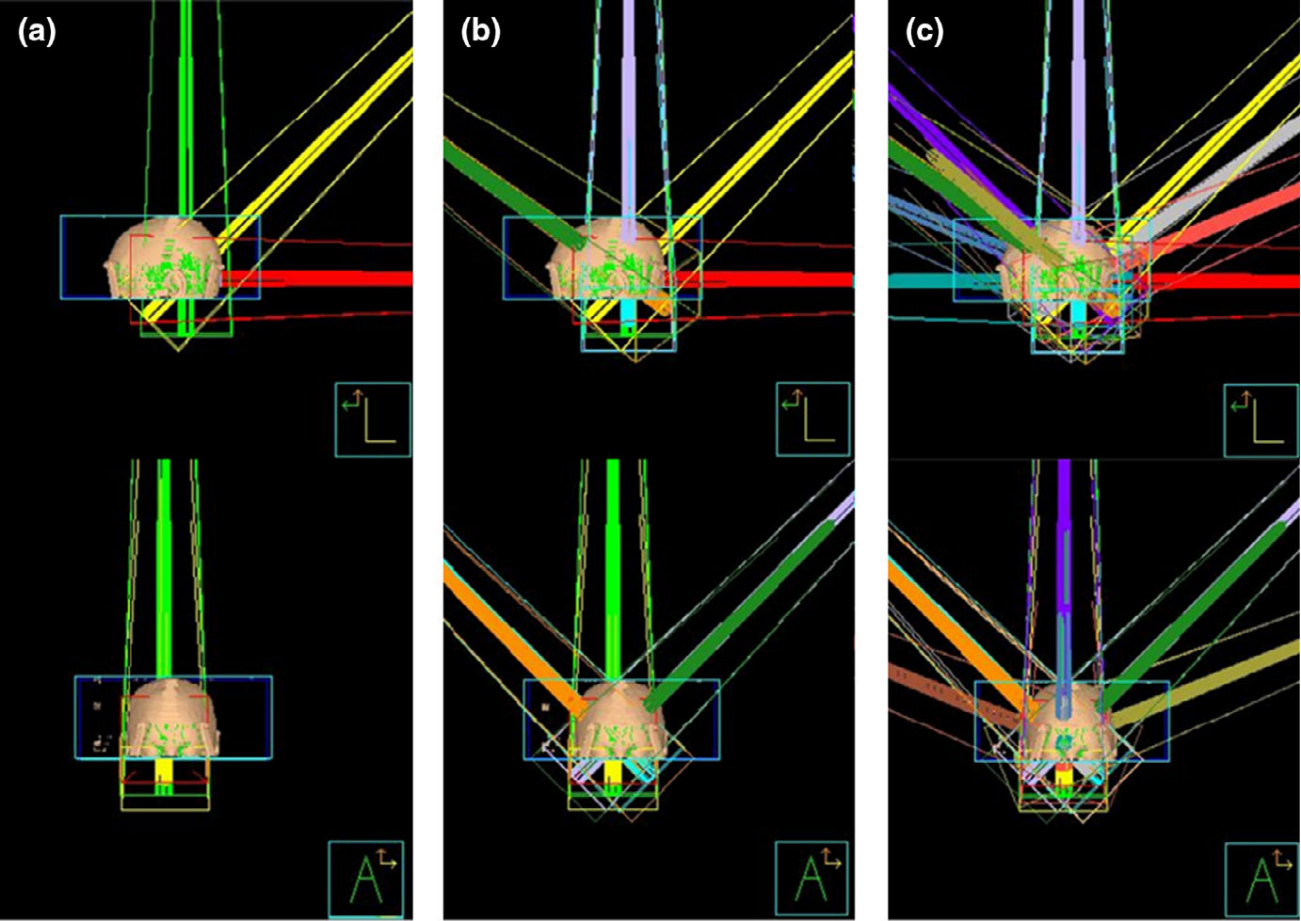
Example shaped beam arrangements for the proton plans. (a) 3 beam (b) 9 beam and (c) 15 beam arrangements. Top image showing a left lateral view and bottom image showing an anterior view

Currently, passive scatter proton treatment plans for fractionated cases are created using 2–4 beams per target to increase conformity, increase robustness, and spread uncertainties due to range, patient setup, and patient motion.^2^ Multiple plans were created to test how many beams were necessary to produce gradient and conformity indices similar to VMAT SRS plans. Seven plans with number of beams ranging from 1 to 21 beams were created. Beams were arranged in fixed beam geometry using similar couch angles and gantry angles that would be used for SRS arc therapy and to mimic the arc rotations used in linac‐based arc SRS plans. Figure 1 shows beam arrangements for 3, 9, and 15 beams.

The last parameter that was evaluated was the use of a compensator to shape the distal edge of the target. In some cases, the use of a compensator could possibly cause an increase in the lateral penumbra and an increase in the air gap required for a plan. For use in this planning study, the compensators were generated using a smearing value of 2 mm and edge processing value of 10 mm. The plan that had the best metrics without a compensator was also compared to a plan with a compensator to match the distal edge and those metrics were also reported. All of the plans were compared to the HyperArc™ plans for the same dataset.

### 2.3 Treatment planning study

Ten patients who had previously been treated with linac SRS were chosen for this study. The patients were selected based on their tumor types, sizes, and complexity. For simplicity, patients having multiple metastases were excluded from the planning study. Table 1 contains the tumor type, size, complexity, and prescription dose of the ten radiosurgery patients selected for the study. The study population consisted of three arteriovenous malformations, three meningiomas, one metastasis, and three vestibular schwannomas ranging in size ranged from 0.3 to 10 cm^3^. *Simple* targets were planned using a single spherical isocenter, and *complex* targets required multiple isocenter plans for adequate conformity.

**Table 1:** Summary of target volume characteristics for the 10 patients included in this study.

Tumor type	Patient	Target volume (cm^3^)	Complexity	Prescription (Gy)	Proton beams
Arteriovenous Malformations	1	5.02	Simple	20	4
	2	8.58	Complex	20	3
	3	10.04	Complex	20	3
Meningioma	4	0.30	Simple	15	3
	5	1.06	Simple	12.5	3
	6	8.15	Complex	20	4
Metastases	7	1.14	Simple	20	4
Vestibular Schwannomas	8	4.57	Simple	12.5	4
	9	1.79	Complex	12.5	5
	10	5.61	Complex	12.5	4

Under the approval of the institutional review board, the CT and MR image data sets and radiation treatment structure sets for each patient were transferred using an encrypted external hard drive and imported into the Pinnacle™ TPS for proton plans and into the Eclipse™ (Varian Medical Systems, Palo Alto, CA) TPS to create HyperArc™ VMAT plans. For simplicity, the surrounding brain was considered normal tissue and no other structures besides the GTV were contoured. The normal tissue contour was created by using Boolean by subtracting the GTV from the whole body contour.

The VMAT plans were created using Eclipse Version 15.6 and using the Varian HyperArc™ planning feature for SRS optimization. This SRS software uses a set combination of arcs and the optimization was performed to minimize normal tissue doses while fixing the coverage such that 98% of the target receives 100% of prescribed dose. For this study, single, solitary targets were chosen such that all plans were designed to a single isocenter and single target. The VMAT plans with HyperArc™ were compared to the PSRS plans using a set of metrics used for SRS plan comparisons and integral dose. The VMAT and PSRS plans were normalized for 98% and 95% coverage, respectively, in keeping with guidelines for both IMRT and proton therapy.^1^, ^11^, ^12^

The proton plans were generated in Pinnacle™ planning system using the findings as shown from the plan parameter optimization. All plans were to a single target using a single isocenter. Beam selection uses specific couch/gantry combinations that would align to standard SRS arc therapy beams. The aperture per beam was auto‐set to 0.5 mm around the target (CTV). A range margin of 2%*Range + 2 mm was used (resulting in proximal and distal margin of approximately 5 mm). Plans were created starting with 3 beams, but in some cases due to irregularly shaped targets more were added (up to 5) as needed to cover the CTV. Beams were placed on the ipsilateral side of the patient. Compensators were added to all fields using a smearing margin of 2 mm and an edge processing margin of 10 mm. Based on the planning parameters, the plans were normalized to an isodose line close to 65% such that at least 95% of the target received the prescribed dose.

#### 2.3.1 Planning metrics

Wagner et al. defines parameters to optimize radiosurgery plans as the Conformity/gradient index (CGI) which is made up of two terms, a conformity score (CGIc), and a gradient score (CGIg) both normalized to a 100‐point scale. Dose conformity is especially important in radiosurgery plans due to the large radiation dose delivered in a single fraction. Wagner et al. defines the conformity score as follows:

(1) $$\text{CGIc}=100\left(\frac{\text{Target}\;\text{Volume}}{\text{Prescription}\;\text{Isodose}\;\text{Volume}}\right)=\left({\text{PITV}}^{‐1}\right)\times 100$$

An ideal conformity score is 100, where 100% of the target volume is covered by the prescription isodose volume. The conformity score is a scaled version of the conformity index defined by the radiotherapy oncology group (RTOG) where PITV is the ratio of prescription isodose volume to target volume.^13^ RTOG states the conformity index should be between 1.0 and 2.0 which corresponds to 100% to 50% for Wagner’s conformity score.^14^ Wagner et al. defines the gradient score as follows:

(2) $$\text{CGIg}=100‐\left(100\left[\left(R_{\text{Eff},50\%RX}‐R_{\text{Eff},Rx}\right)‐0.3\text{cm}\right]\right)$$

where R_Eff,Rx_ is the effective radius of the prescription isodose volume and R_Eff,50%Rx_ is the effective radius of the isodose line that is equal to one‐half the prescription isodose volume. The gradient score is a measure of how sharply the dose falls off, such that a score greater than or equal to 100 corresponds to an optimum 3 mm or less gradient.^13^, ^14^ The final parameter CGI is defined by averaging CGIg and CGIc as shown in Eq. (3). A plan with ideal coverage (PITV = 1 and an ideal gradient of 3 mm) would give a CGI score of 100.

(3) $$\text{CGI}=\left(\text{CGIg}+\text{CGIg}\right)/2$$

The homogeneity index (HI) is defined by the RTOG as the ratio of the maximum dose to the prescription dose. The HI is used to describe the uniformity of dose within the target.^13^, ^14^ The final parameter is integral dose (ID) is shown in Eq. (4) which is defined as the mean dose (Gy) multiplied by the volume (cm^3^) of a structure. This quantity is used to assess irradiation of healthy tissue volumes (brain volume subtracted from target volume).

(4) ID=MeanDose×Volume

In addition to the parameters explained above, each plan’s dose volume histogram (DVH) was analyzed to compare target coverage and normal tissue dose. The target volume receiving 95% of the dose (V_95_), the mean dose within the target volume (D_mean_), the minimum dose delivered to the target volume (D_min_), the volume of normal brain tissue receiving 12 Gray (Gy) (V_12Gy_), and a normalized volume of normal brain tissue receiving 12 Gy (V12_GyNorm_) were calculated.^1^, ^15^, ^16^, ^17^, ^18^, ^19^, ^20^ The V12_GyNorm_ was normalized to each patient’s target volume for a straightforward comparison.

## 3 RESULTS

### 3.1 Verification of TPS dosimetry for small fields

**Fig. 2:**
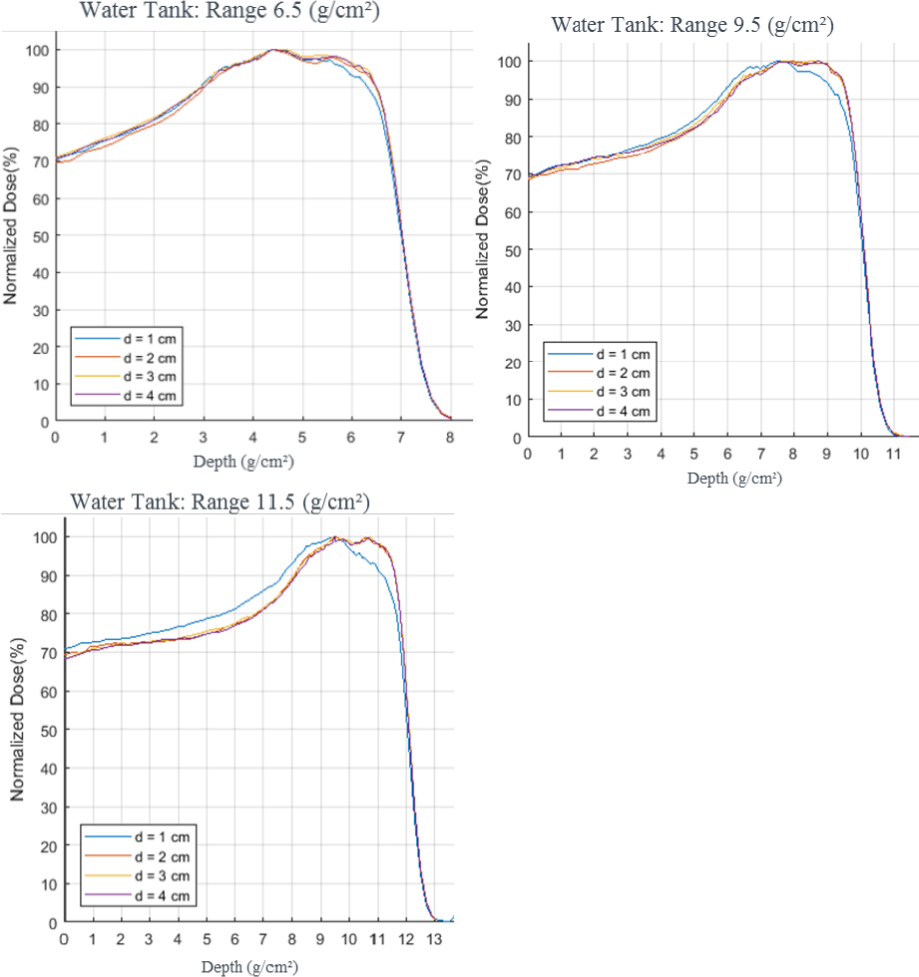
Water tank depth dose measurement results comparing different aperture sizes for varying proton ranges, all measurements used a modulation of 3 cm d’ refers to the diameter opening of the aperture used

Tables 2, 3, 4 outline the range, distal falloff, and modulation results comparing in‐water, film, and TPS measurements, respectively. For each range, d’_90_ increased as the aperture size increased. The range of d’_90_ was within 1 mm of the expected range for all measurements and for the treatment plans. The DDF for the 1 cm diameter aperture for each range was the largest and the 2, 3 and 4 cm apertures produced similar values for DDF. As the range increased, the discrepancy between the 1 cm DDF and the remaining aperture’s DDF increased. For example, for the in‐water measurements, the difference between the 1 cm and 2 cm DDF was 0.0 g/cm^2^, 0.05 g/cm^2^, and 0.11 g/cm^2^ for ranges 6.5 g/cm^2^, 9.5 g/cm^2^, and 11.5 g/cm^2^, respectively. This trend was seen in each of the film, water, and TPS depth measurements. This is because as the energy increases, the degradation in the distal portion of the SOBP becomes more evident because the loss of electronic equilibrium due to multiple Coulomb scattering (MCS).^9^ Figure 2 shows the in‐water WT (water tank) SOBP results graphically, using a modulation of 3 cm, for (a) range 6.5 g/cm^2^, (b) range 9.5 g/cm^2^, and (c) range 11.5 g/cm^2^ for the four different field sizes.

**Table 2:** Proton range (d’_90_) in (g/cm^2^) for in‐water measurements, film measurements, and TPS calculations compared to Nominal Range.

Aperture diameter (cm)	6.5 (g/cm^2^)			9.5 (g/cm^2^)			11.5 (g/cm^2^)		
	In‐water	Film	TPS	In‐water	Film	TPS	In‐water	Film	TPS
1	6.31	6.19	6.49	9.31	8.96	9.41	11.1	10.85	11.3
2	6.47	6.26	6.53	9.57	9.17	9.51	11.6	11.05	11.4
3	6.48	6.41	6.52	9.56	9.29	9.51	11.6	11.10	11.4
4	6.48	6.40	6.52	9.60	9.31	9.51	11.6	11.18	11.4

**Table 3:** Distal dose fall‐off (DDF) in cm for in‐water measurements, film measurements, and TPS calculations for different proton ranges.

Aperture Diameter (cm)	6.5 (g/cm^2^)			9.5 (g/cm^2^)			11.5 (g/cm^2^)		
	In‐water	Film	TPS	In‐water	Film	TPS	In‐water	Film	TPS
1	0.70	0.84	0.70	0.70	0.87	0.71	0.75	1.11	0.76
2	0.66	0.81	0.68	0.65	0.82	0.67	0.64	0.81	0.70
3	0.65	0.76	0.68	0.65	0.82	0.67	0.64	0.84	0.69
4	0.66	0.82	0.68	0.64	0.81	0.67	0.64	0.94	0.69

**Table 4:** Modulation (m’_95_) in cm for in‐water measurements, film measurements, and TPS calculations for different proton ranges.

Aperture Diameter (cm)	6.5 (g/cm^2^)			9.5 (g/cm^2^)			11.5 (g/cm^2^)		
	In‐water	Film	TPS	In‐water	Film	TPS	In‐water	Film	TPS
1	3.37	3.57	3.25	3.58	3.66	3.25	3.50	4.20	3.28
2	3.46	3.61	3.23	3.65	3.99	3.21	3.50	4.22	3.19
3	3.54	3.81	3.22	3.64	4.35	3.19	3.56	4.41	3.18
4	3.47	3.59	3.22	3.61	3.97	3.19	3.45	3.85	3.18

When comparing the individual modulation measurements with different apertures, the film results for m’_95_ showed the same trends as the range. The values increased as the aperture diameter increased until there was an unexpected decrease in the 4 cm diameter aperture. This trend was also evident in the WT SOBP measurements but not the TPS simulated measurements. This is potentially associated with MCS, since the 4 cm diameter field size may be large enough to not be affected by a loss of electronic equilibrium. The m’_95_ for the TPS showed little variation with field size.

**Fig. 3:**
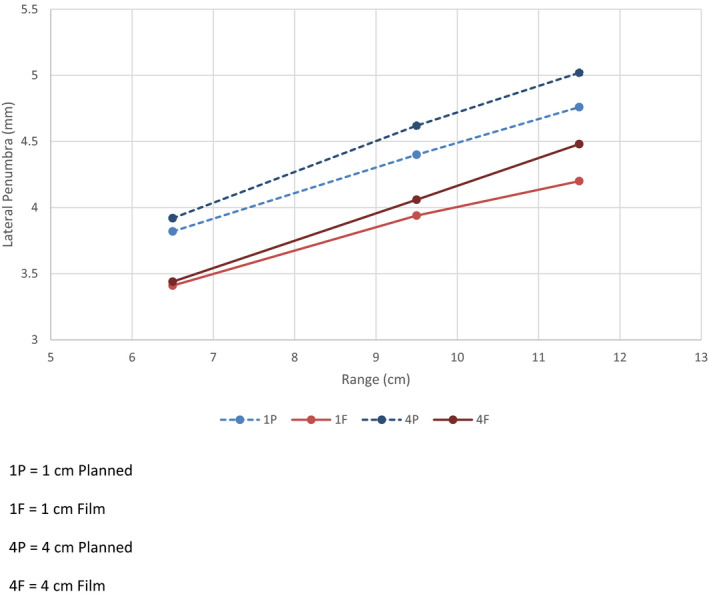
Comparison of lateral penumbra as a function of proton range comparison between film and treatment planning system

Table 5 shows the LP results comparing the film measurements to the treatment plans for the various ranges and field sizes. The average LP was calculated for both the film measurements and the treatment plans by averaging the penumbra at FWHM on each side of the beam profile taken in the middle of the SOBP. For each range, there was no significant difference when comparing the lateral penumbra values and aperture diameter. However, the LP did increase with increasing range, which is expected due to the increasing energy. The lateral penumbra vs range and field size was plotted and shown in Fig. 3 for the TPS (dashed) compared to film (solid) for both 1 and 4 cm apertures. For all field sizes there was a difference between the TPS and the film where the TPS overestimated the LP by approximately 0.5 mm.

**Table 5:** Film clinical setup experiment beam profile results.

Aperture Diameter (cm)	Lateral Penumbra (cm): Film			Lateral Penumbra (cm): TPS		
	R6.5M3	R9.5M3	R11.5M3	R6.5M3	R9.5M3	R11.5M3
1	0.34	0.39	0.42	0.36	0.33	0.33
2	0.33	0.41	0.46	0.35	0.36	0.36
3	0.35	0.40	0.46	0.37	0.35	0.35
4	0.34	0.41	0.45	0.36	0.37	0.37

### 3.2 Optimizing PSRS planning parameters

**Fig. 4:**
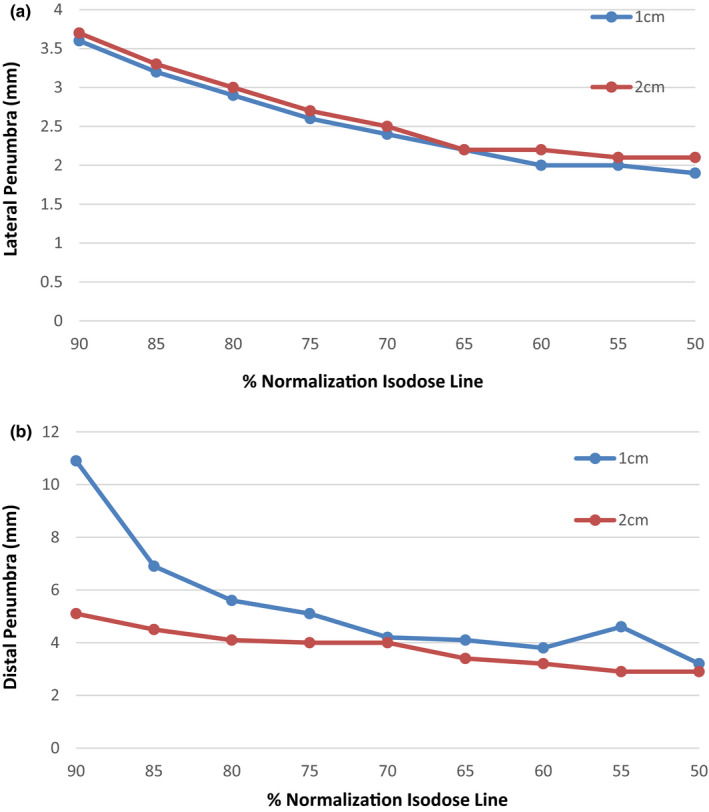
(a) and (b) Lateral and distal penumbra as measured between Prescription Isodose line and ½ of the Prescription isodose line for various isodose lines. (All lines are normalized to Max Dose)

Plotted in Figs. 4(a) and 4(b) are the lateral (4a) and distal (4b) penumbra vs normalization isodose line. Based on these figures an optimal prescription isodose line that yields the sharpest lateral and distal penumbra is approximately the 65% isodose line.

**Fig. 5:**
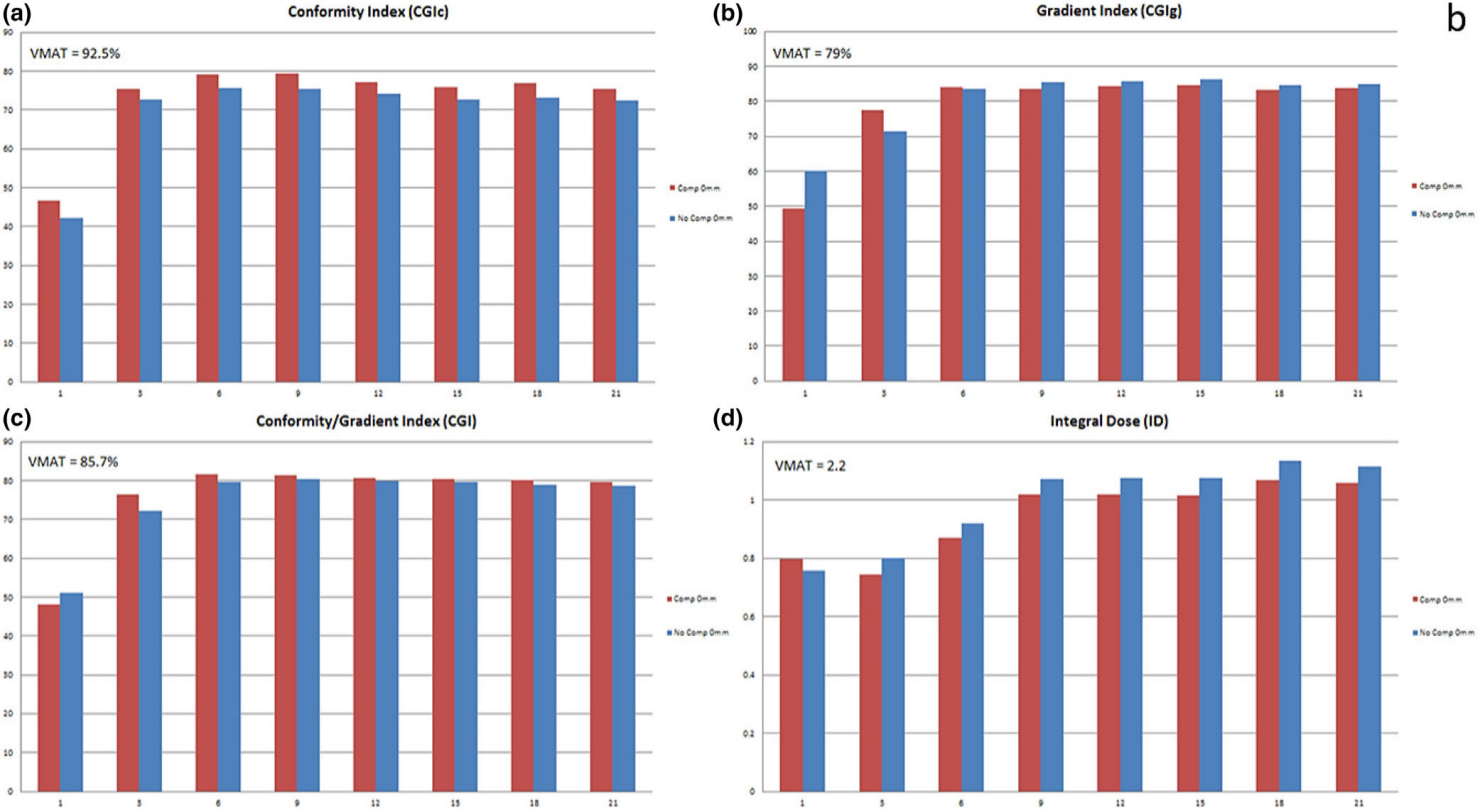
Number of beams experiment with & without compensators, comparing CGIc (a), CGIg (b), CGI (c), and ID (d)

**Fig. 6:**
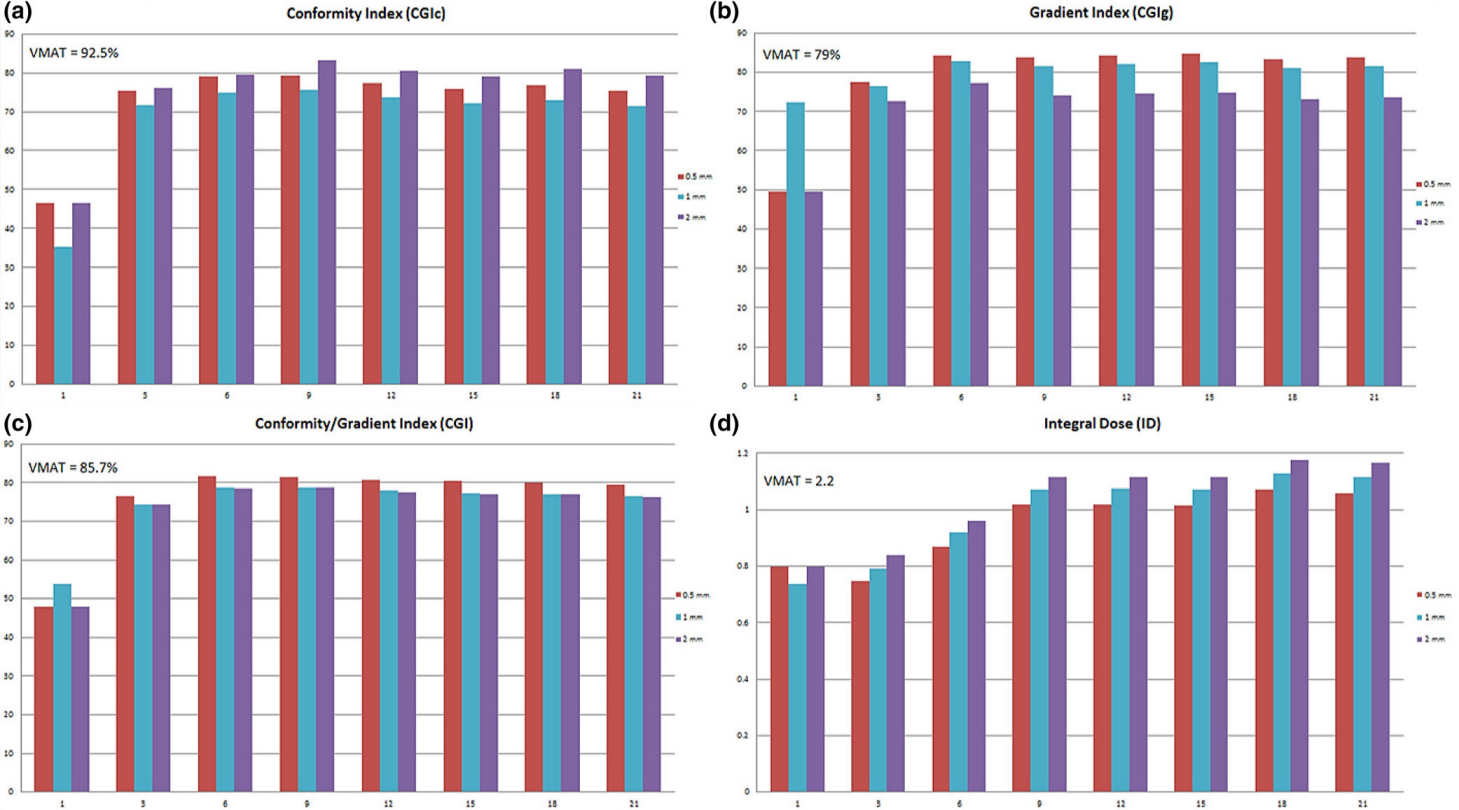
Number of beams vs aperture margin, comparing CGIc (a), CGIg (b), CGI (c), and ID (d)

Figure 5 shows the comparison between the proton plans created with and without compensators for different numbers of proton beams on a single test patient plan. When looking at CGIc, Fig. 5(a), the proton plans created with a compensator are superior for all beam arrangements. However, the compensator plans still failed to match the HyperArc™ plan for this same patient (given as VMAT values in the graph). Figure 6 compares various aperture margins and numbers of beams with each plan normalized to an isodose line to achieve similar coverage. For conformity and gradient index which is a measure of both conformity and gradient, the best plans were achieved using the 0.5 mm margin apertures. When evaluating the number of beams needed both Figs. 5 and 6 indicate that the optimal number of proton beams is between 3 and 6 beams.

In all cases, the ID was less for proton plans, even with 21 proton beams, compared to HyperArc™ plans, but the ID also did not increase for proton plans beyond 9 beams due to the overlap of the distal edge of the proton beams above 9 beams. With nine beams or greater, the proton plans have an ID of less than 1.1 compared to the VMAT plan ID of 2.2. Also, for plans with three beams or more, the plans with compensators have a lower ID than plans without compensators due in part to the increased conformality at the distal edge of the beam which limits the high dose received to the normal brain. On average, the compensator plans provided 8% less ID when compared to the non‐compensator plans primarily due to the conformality of the distal edge of the beam to the target. Due to these results and the current treatment plans for passive scatter proton units, it is suggested to use 6 beams or less per treatment.

Based on findings on the simple case, it was determined that using a 0.5 mm lateral aperture margin provided plans that can be normalized to roughly 65–70% isodose lines. Plans were created on the ten different test cases and normalized such that at least 95% of the target was covered by the prescription isodose line. Additionally, it was determined that the optimal number of beams is between 3‐6. The number of beams used per target is show in Table 1.

### 3.3 Planning comparison

Table 6 shows the results of conformity and gradient calculations for both HyperArc™ and proton plans. The HyperArc™ plans had mean CGIc, mean CGIg, and mean CGI of 90.9 ± 11.3, 84.3 ± 9.6, and 87.6 ± 8.1, respectively. The proton plans had a mean CGIc, a mean CGIg, and a mean CGI of 67.3 ± 8.5, 83.9 ± 6.3, and 75.6 ± 5.0, respectively. Overall, the HyperArc™ plans achieved superior conformity compared to the proton plans. With respect to the gradient the proton plans and the HyperArc™ plans were very similar.

**Table 6:** Results of target volume conformity and gradient metrics.

Patient	Conformity index		Gradient index		Conformity gradient index	
	VMAT	Protons	VMAT	Protons	VMAT	Protons
1	93.8	80.5	82.9	81.8	88.4	81.1
2	83.8	60.9	69.0	75.9	76.4	68.4
3	86.4	67.4	67.8	80.1	77.1	73.8
4	63.0	55.3	91.5	85.1	77.3	70.2
5	96.2	68.2	93.3	96.0	94.8	82.1
6	96.4	79.4	82.8	76.5	89.6	78.0
7	102.8	67.3	95.9	91.9	99.3	79.6
8	90.5	70.8	88.1	86.5	89.3	78.6
9	97.9	55.5	89.9	84.0	93.9	69.8
10	97.9	68.0	82.0	81.4	89.9	74.7
Mean ± SD	90.9 ± 11.3	67.3 ± 8.5	84.3 ± 9.6	83.9 ± 6.3	87.6 ± 8.1	75.6 ± 5.0

Table 7 outlines the target volume dose coverage for each of the different plans. For comparison purposes, the values for V_95%,_ D_mean_, and D_min_ were each normalized to the prescription dose. By design the VMAT plans were created such that 98% of the target is covered by the prescription line so the V95% was always 100%. The coverage in the proton plans was on average 98%. The mean doses for the VMAT plans averaged 114% higher than prescription dose compared to the proton plans whose average dose was 130% compared to prescription dose. The HI for the VMAT and proton plans were both within recommendations (<2) for SRS, with the VMAT plan average HI of 1.28 and the proton plan HI of 1.5.

**Table 7:** Results of target volume parameters normalized to the prescription dose.

Patient	V_95%_ (%)		D_mean_ (%)		D_min_ (%)		Homogeneity index	
	VMAT	Protons	VMAT	Protons	VMAT	Protons	VMAT	Protons
1	100.0	96	115.2	125.4	92.1	64.6	1.30	1.43
2	100.0	98	112.9	136.3	91.0	62.4	1.24	1.54
3	100.0	98	106.3	130.3	93.0	34.6	1.21	1.43
4	100.0	100	106.6	121.0	98.0	86.8	1.14	1.41
5	100.0	98	117.0	129.6	93.3	74.7	1.35	1.53
6	106.0	99	115.8	125.7	89.7	77.3	1.33	1.43
7	100.0	97	117.3	130.3	94.6	70.0	1.34	1.53
8	100.0	99	117.9	135.3	93.0	79.2	1.30	1.53
9	100.0	98	115.6	134.5	86.2	56.1	1.30	1.61
10	100.0	99	113.3	135.3	91.7	67.0	1.28	1.53
Mean ± SD	100.6 ± 1.8	98.2 ± 1.1	113.8 ± 4.0	130.4 ± 4.9	92.3 ± 2.9	67.3 ± 13.8	1.3 ± 0.1	1.5 ± 0.1

Normal tissue and integral dose are shown in Table 8. The ID calculations for the proton plans were on average 2 times lower when compared to the VMAT plans. The V_12Gy_ volumes were all less for the VMAT plans when compared to the proton plans. The proton plans exceeded the photon VMAT plans ranging from a 22% ‐ 72% increase in normal tissue volumes receiving 12 Gy.

**Table 8:** Results of normal tissue parameters.

Patient	Integral Dose (Gy)			V_12Gy_ (cm^3^)		V_12GyNorm_ (cm^3^/ cm^3^)	
	VMAT	Protons^a^	VMAT/Protons	VMAT	Protons*	VMAT	Protons
1	2.16	0.79	2.73	7.26	9.2	1.45	1.83
2	3.05	1.42	2.15	15.71	20.52	1.90	2.39
3	3.22	0.99	3.25	17.64	19.03	1.77	1.90
4	0.35	0.14	2.57	0.81	0.96	2.79	3.18
5	0.35	0.12	2.98	0.84	0.66	0.82	0.62
6	1.50	0.77	1.96	9.54	14.91	1.19	1.83
7	0.98	0.30	3.33	1.92	3.32	1.75	2.91
8	1.14	0.50	2.28	0.88	2.43	0.20	0.53
9	0.86	0.39	2.18	0.30	1.79	0.17	1.05
10	1.39	0.65	2.14	0.77	3.33	0.14	0.59

^a^Proton plans normalized to 65–70%.

## 4 CONCLUSION

### 4.1 Verification of TPS dosimetry for small fields

The literature has quoted typical specifications for a passive scatter beam line for d’_90_ (specified in water) to be within ± 1 mm of the requested range. In this study, for field sizes > 10 mm, the in‐water and TPS measurements were in good agreement and were within 1 mm of specified range. For the 10 mm aperture, d’_90_ measurements for all three ranges agreed between TPS and water measurements, but were proximal by more than 1 mm than the expected range. This is due to the fact that as the field size decreases the loss of charged particle equilibrium increases and results in a deterioration of the Bragg peak and non‐uniformity of SOBP.^21^, ^22^ What is important is that the planning system properly accounts for this change in range with small fields.

Penumbra measurements for the planning system and film were in good agreement. The DDF for double scatter has been reported to be 0.70 g/cm^2^.^21^, ^22^ In this study, the DDF for small fields were in good agreement between TPS and measurements. The current study found that for proton ranges 6.5 g/cm^2^, 9.5 g/cm^2^, and 11.5 g/cm^2^ the average LP between all aperture sizes were 3.4, 4.0, and 4.2 mm, respectively. Rana et al.^23^ found the LP for proton ranges 8 g/cm^2^, 10 g/cm^2^, and 12 g/cm^2^, for a scanning proton beam, were 5, 5.2, and 5.4 mm, respectively and showed similar difference in LP between TPS and measurements while using a different TPS. The passive scatter beam provides a sharper penumbra due to the added collimator used during treatment. When comparing the results to linac‐based SRS, Heydarian et al.^24^ found that for a 7 mm and a 23 mm circular collimator the LP was 2.65 and 3.20 mm, respectively. This study showed for 10 and 20 mm apertures the LP was 3.4 and 3.3 mm for a 6.5 g/cm^2^ range, 3.9 and 4.1 mm for a 9.5 g/cm^2^ range, and 4.2 and 4.6 mm for an 11.5 g/cm^2^ range^25^.

The LP for charged particles increases as the range increases because the particle experiences MCS. Every time a particle has an interaction it is deflected by a very small angle. This effect of deflection accumulates more and more as the particle’s range increases and causes the particle to spread laterally (i.e., the LP increases). All the while, the particle energy decreases and the deflection angle increases for each interaction causing the LP to increase faster near the end of the beam range.^21^, ^22^, ^26^ Overall, the small field dosimetry showed adequate agreement between the TPS and measured data.

### 4.2 Optimizing PSRS planning parameters

Based on the plans created on a single metastatic SRS lesion the optimal plan parameters were determined. This includes using a very tight margin of 0.5 mm to define the aperture, use of compensators to better shape distally, and between 3 and 6 beams.

### 4.3 Planning comparison

**Fig. 7:**
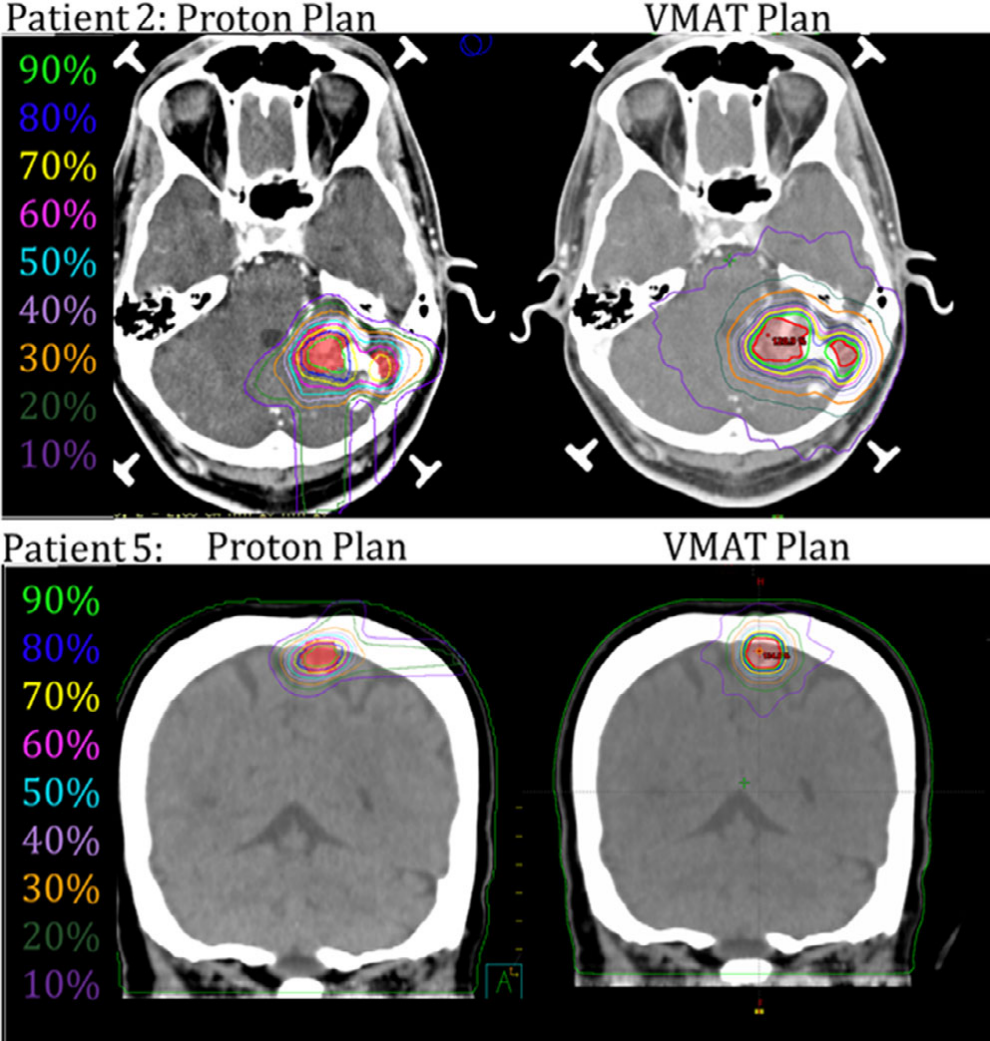
Isodose lines comparing two different planning techniques for Patient 2 and Patient 5

**Fig. 8:**
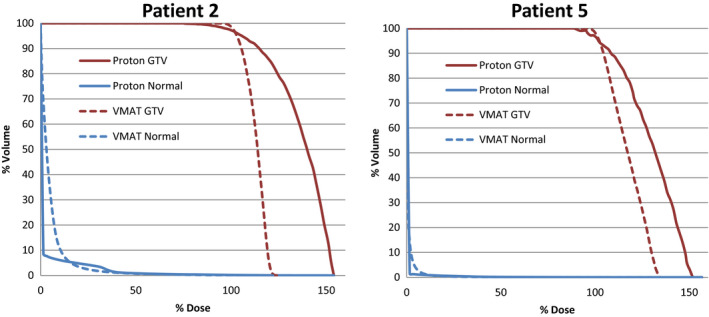
Dose volume histogram (DVH) for GTV and Normal tissue comparing volumetric modulated arc therapy stereotactic radiosurgery (SRS) to PSRS

Wagner et al. found that a CGIg scores of ≥90 are achievable for small targets with simple geometries and a CGIg score range of 60 ‐ 80 for more complex cases. Patients 1, 4, 5, 7, and 8 were categorized as simple. Patients 1 and 8 were unable to meet Wagner’s quoted CGIg score of ≥90 for the VMAT plans, while none of the proton plans achieved a CGIg score of ≥90. For the complex categorized patients, all of the VMAT and proton plans met Wagner’s quoted CGIg range of 60–80. When comparing each patient individually, 7 patients planned using protons performed within 10% of the VMAT plans when comparing CGIg scores. The RTOG guidelines state plans should have a minimum CGIc score of 50, with an ideal CGIc score of 100.^13^ All of the patients from both VMAT and proton plans pass the minimum score of 50, while Patients 4, 5, 7, and 8 scored 100 or greater for the VMAT plans. When comparing each patient individually, 8 patients planned using protons performed within 15% of the VMAT plans when comparing CGIc scores. The VMAT plans are more conformal by nature and the proton plans had difficulty competing. Figure 7 shows two patient treatment plans comparing the proton and VMAT dose distributions and Fig. 8 shows the DVH for the VMAT and proton plans for the same patients. Patient 2 clearly shows how protons are at a disadvantage when target volumes are irregularly shaped. Patient 5 shows highly conformal dose distributions for all three plans. However, the proton plan shows the lower dose isodose lines to be more tightly conformed to the target, leading to a lower integral dose to surrounding normal tissues.

Halasz et al., found a median CGIg of 81.7, and a median GCIc of 45.5 while comparing 50 meningioma treatments using a passive scatter proton unit.^7^ The research conducted for this study found a median CGIg of 75.0, and a median CGIc of 85.5. The proton plans created for this research conformed to the target approximately 45% more but had a lower gradient score by approximately 8%.

For small intracranial targets, Bolsi et al. showed that using passive scattering proton beams rather than stereotactic arc therapy reduced the integral dose from 9.3 to 3.2 Gy*cm^3^/10^3^, corresponding to approximately 3 times lower for the proton plans.^8^ This study showed an average of 2 times lower ID when comparing the proton plans to the VMAT plans. ID has been a leading factor in propelling the use of proton radiation therapy. A lower ID may be especially important in patients with benign tumors who generally have long life expectancy and thus may be more likely to experience radiation related late effects, including hypopituitarism and secondary cancers.^9^

For special cases, such as pediatric or retreated patients, it is crucial for the radiation dose to normal tissues to be as low as possible, and proton radiation therapy has proven it is a great alternative to traditional treatments. It has been demonstrated that lesions treated with V_12Gy_> 8–10 cm^3^ have more than a 10% risk of radionecrosis and should be considered for hypofractionated stereotactic radiotherapy, especially when located in/near eloquent areas.^18^, ^19^, ^24^ Patients 2, 3, and 6 had TVs of 8.2 cm^3^, 10 cm^3^, and 10 cm^3^, respectively. Since these TVs are in the quoted range of > 10% risk of radionecrosis, they were excluded from the further comparison. Even though the VMAT plans provided overall lower 12 Gy volumes, only one of the proton patients failed to meet the 8–10 cm^3^ range for increased risk of radionecrosis with a V_12Gy_ of 10.88 cm^3^. As expected, patients treated with a higher prescription dose had a larger volume receiving 12 Gy.

## 5 DISCUSSION

The commercial planning system (Pinnacle™) shows favorable findings in how the planned dose distribution compare to delivered for small field double‐scatter proton beams. Additional work should be performed into the other dosimetry aspects of beam output and absolute dose if small fields are to be used for treatment, but these data indicate that the plans generated for this study are appropriate for plan comparisons.

The data presented in this study have shown the HyperArc™ plans are superior to proton plans, except as it relates to the integral dose, where in most cases the proton therapy gives less integral dose. A few limitations were inherent in the proton planning stage. First, the proton plans were created using forward planning and using conformal beams. The HyperArc™ plans will always provide higher conformity because of the modulated arcs used to treat the target. Larger margins can be added to the range or lateral edge of the target to increase conformity of the dose distribution.

Another caveat with the proton planning is due to the range uncertainty. Range uncertainty for the plans in this planning exercise was on the order of 5 mm. Range uncertainty is a function of the range of the proton beam, the heterogeneity of the path of the proton beam, and also the level of certainty the institution has within their CT scanner values and their corresponding stopping power. With dual energy CT scanning and accurate CT to stopping power measurements, it is possible to minimize this uncertainty, especially in areas that are heterogeneous and shallow, as is used in ocular proton treatments. With decreased range uncertainty, it may be possible to treat targets much deeper in the brain, however, based on clinical range uncertainties used for our facility and our treatment machine, the conformity of the prescription line was worse for small targets (<10mm) with protons compared to photons. Additional limitations to small targets in this study was the minimum modulation of 2 cm for the Mevion Proton machine increases this range and modulation margin even more for very small targets. For this study, the smallest dimension of any target was 8 mm and in that plan comparison the proton plan metrics were worse than the VMAT plan for all aspects of the plan.

Finally, the proton plans were created using forward planning. All treatment planning is dependent on the expertise of the planner and the technology available for optimization and planning. With that being said, the treatment plans created in this study can potentially be improved. However, even with the limitations, the proton plans performed within quoted guidelines and limits suggested by literature and were far superior when analyzing the integral dose values.

One other consideration is the time required to deliver the proton plans compared to conventional SRS or to HyperArc™ plans is still greater. Based on our experience if we assume imaging time to be the same between a Linac room and a Proton room, the time to deliver a high dose HyperArc™ treatment is approximately 5‐10 min with dose rates on the order of 2000 cGy/min, whereas for the current double scattered proton therapy system, the dose rate is on the order of 200 cGy/min requiring approximately 5 min per beam or treatment times of at least 30–40 min. While this is longer than for the Linac‐based SRS, it is assumed that a proton SRS treatment could be given within a 1 h treatment session and should not be the only thing to consider when deciding the appropriate treatment for a patient.
